# Potential Correlation Between Molecular Biomarkers and Oxidative Stress in Traumatic Brain Injury

**DOI:** 10.3390/ijms26083858

**Published:** 2025-04-18

**Authors:** Cătălina Ionescu, Madalina Ghidersa, Alin Ciobica, Ioannis Mavroudis, Dimitrios Kazis, Foivos E. Petridis, Dragoș Lucian Gorgan, Ioana-Miruna Balmus

**Affiliations:** 1 Department of Biology, Faculty of Biology, “Alexandru Ioan Cuza” University of Iasi, 700505 Iasi, Romania; catalinaionescu81@yahoo.com (C.I.); madalinamghidersa@gmail.com (M.G.); alin.ciobica@uaic.ro (A.C.); lucian.gorgan@uaic.ro (D.L.G.); 2 “Ioan Haulica” Institute, Apollonia University, 700511 Iasi, Romania; 3 Center of Biomedical Research, Romanian Academy, Iasi Branch, 2 Teodor Codrescu Street, 700481 Iasi, Romania; 4 Academy of Romanian Scientists, 050094 Bucharest, Romania; 5 Department of Neurosciences, Leeds Teaching Hospitals, Leeds LS9 7TF, UK; 6 Third Department of Neurology, Aristotle University of Thessaloniki, 541 24 Thessaloniki, Greece; dimitrios.kazis@gmail.com (D.K.); f_petridis83@yahoo.gr (F.E.P.); 7 Department of Exact Sciences and Natural Sciences, Institute of Interdisciplinary Research, “Alexandru Ioan Cuza” University of Iasi, 700057 Iasi, Romania; balmus.ioanamiruna@yahoo.com

**Keywords:** traumatic brain injury, biomarkers, oxidative stress, diagnosis, prognosis

## Abstract

Diagnosing traumatic brain injury (TBI) remains challenging due to an incomplete understanding of its neuropathological mechanisms. TBI is recognised as a complex condition involving both primary and secondary injuries. Although oxidative stress is a non-specific molecular phenomenon observed in various neuropathological conditions, it plays a crucial role in brain injury response and recovery. Due to these aspects, we aimed to evaluate the interaction between some known TBI molecular biomarkers and oxidative stress in providing evidence for its possible relevance in clinical diagnosis and outcome prediction. We found that while many of the currently validated molecular biomarkers interact with oxidative pathways, their patterns of variation could assist the diagnosis, prognosis, and outcomes prediction in TBI cases.

## 1. Introduction

Traumatic brain injury (TBI) is a leading cause of disability and progressive cognitive decline following head trauma [1,2]. The interplay between primary and secondary brain injuries contributes to the multiple-hit hypothesis, which suggests that cumulative effects of trauma lead to disrupted brain function [1,3]. The short-term effects of TBI vary depending on the type of trauma and the brain regions affected, with common symptoms including headaches, aphasia, amnesia, and seizures [2]. Additionally, cognitive impairment, mood disturbances, and motor deficits may persist for months or even years post-injury [3].

Mechanical impact on the skull can result from various types of traumas, influencing the brain’s response to injury. TBI can occur due to motor vehicle accidents, falls, or blunt force impacts, including head-to-head collisions. Similarly, sports-related injuries, particularly in contact sports such as soccer, rugby, and American football, are common causes of TBI. Certain occupations also carry an elevated risk of TBI due to exposure to blasts, violence, falls, or blunt trauma—these include military service, law enforcement, and construction work.

Head trauma is classified into three main types based on the mechanism of injury: closed-head trauma, penetrating trauma, and explosive blast trauma. Each mechanism results in distinct types of damage, clinical outcomes, and biomarker involvement [2,4,5,6,7]. Closed-head trauma does not involve skull penetration; however, the transmitted force can cause compression of brain tissues at the impact site, leading to damage to the brain’s vasculature and neurons. In contrast, explosive blast trauma generates extreme kinetic energy, which propagates through the skull, diffuses through brain tissue, and induces deformation. Penetrating trauma, on the other hand, results in focal damage to the microvascular and functional brain tissue near the entry site, increasing the risk of infection and long-term neurological consequences [2,3,5].

The immediate biological response to TBI activates inflammatory and oxidative processes aimed at containing and repairing the damage [2,8]. This pathophysiological cascade underlies the therapeutic success of hypothermic treatment, which helps reduce inflammation preceding decompressive surgery [9]. Many pharmacological strategies target inflammation and oxidative stress modulation. For example, sodium pyruvate is currently used to reduce cortical cell loss, while ethanol pyruvate has been shown to promote neuronal survival [8]. Additionally, monocarboxylates and ketones are often employed as neuroprotective agents [8].

One of the major challenges in clinical practice is the accurate assessment of TBI severity and the prediction of individualised patient outcomes. Current prognostic models rely on demographic variables (e.g., age, sex), clinical indicators (e.g., Glasgow Coma Scale, pupillary response, and associated injuries), and neuroimaging findings (typically from computed tomography scans) [5,10]. While existing protocols primarily emphasise cognitive and motor assessments alongside imaging [11], molecular biomarkers are emerging as valuable tools for improving diagnosis, grading severity, monitoring progression, and predicting prognosis and recovery. Several biomarkers have been validated for clinical use, including neuron-specific enolase (NSE), myelin basic protein (MBP), S100 beta (S100B), glial fibrillary acidic protein (GFAP), and ubiquitin C-terminal hydrolase-L1 (UCH-L1). Although these biomarkers are not TBI-specific, they are widely used in emergency and critical care settings to assess brain injury severity [10].

This review aims to explore the interplay between currently validated TBI biomarkers and oxidative stress pathways, offering new insights into their potential clinical applications. By integrating research and clinical perspectives, we seek to enhance the understanding of TBI pathophysiology and refine diagnostic and prognostic strategies for improved patient outcomes.

## 2. Oxidative Stress in Traumatic Brain Injury

Following head trauma, reactive oxygen species (ROS) are overproduced, and they accumulate in damaged and surrounding tissues, often exceeding the capacity of metabolic and restorative processes to neutralise them, thereby exacerbating tissue damage [12,13,14]. Mitochondria and neutrophils are the primary sources of ROS following TBI [13], alongside other cellular mechanisms such as glutamate-mediated excitotoxicity, NADPH oxidation-reduction reactions, calcium ion release, and catecholamine oxidation [14,15]. The bradykinin pathway also plays a significant role in TBI-associated oxidative stress by modulating phospholipase A2 activation, leading to the release of arachidonic acid, a major precursor of lipid peroxides [16].

Among lipid peroxides, 4-hydroxynonenal (4HNE), isoprostanes (ISOPs), and malondialdehyde (MDA) have been identified as the most abundant oxidative by-products in TBI [16]. The brain, being highly lipid-rich and one of the body’s primary oxygen-consuming organs, is particularly vulnerable to oxidative damage. For instance, 4HNE is derived from n-6-polyunsaturated fatty acids, including arachidonic and linoleic acids, making it a key mediator of oxidative stress in neuronal tissue [16].

Lipid peroxides produced in various tissues are implicated in oxidative stress-induced apoptosis and mitochondrial dysfunction [17]. The presence of ISOPs and MDA in brain tissue signals oxidative damage due to excessive ROS production, while serum MDA primarily reflects cell membrane peroxidation [18,19]. However, because oxidative stress is a hallmark of various neurological and neuropsychiatric disorders, identifying specific oxidative stress biomarkers for TBI remains challenging. Nonetheless, analysing their patterns of variation may provide valuable insights into TBI prognosis and outcomes.

Mitochondria play a dual role in TBI—they are both a source and a target of oxidative stress. Neuronal injury disrupts mitochondrial integrity, leading to leakage and excessive ROS production [13,20]. ROS accumulation extends beyond mitochondria to extra-mitochondrial and extracellular environments, further amplifying oxidative damage. Additionally, oxidative stress has been shown to damage mitochondrial DNA, which lacks histone protection, making it particularly susceptible to mutations that impair ATP synthesis and ROS regulation [15,21].

Due to the brain’s high oxidative metabolic activity and inherently weak repair mechanisms, neurons exhibit exceptional vulnerability to oxidative stress [15]. This susceptibility is particularly evident in selective brain regions, such as the hippocampus, which plays a crucial role in memory and cognition [22]. Interestingly, recent studies suggest that hippocampal neurons can tolerate hypoxia by generating energy independently of oxygen, through anaerobic processes like glycolysis [23].

Astrocytes contribute significantly to neuronal redox homeostasis, irrespective of the source of oxidative stress [24]. They achieve this by neutralising excess extracellular potassium ions and glutamate radicals, using them as substrates for ATP synthesis and mitigating ROS-induced mitochondrial dysfunction. However, a single mild TBI event can trigger progressive astrogliosis [25]. More concerningly, repeated mild TBIs induce a prolonged astrocytic response, persisting for up to six months, and are associated with cognitive impairment [25].

Under certain conditions, astrocytes can also exhibit pro-oxidant and neurotoxic properties, depending on injury severity. They become significant ROS producers and act as microglial activators, further propagating inflammation [26]. Microglia, the brain’s primary immune cells, are among the first responders following TBI, engaging in debris clearance, neuroprotection, and tissue repair [27]. Like astrocytes, microglia also modulate inflammatory responses, promoting the release of pro-inflammatory cytokines, including IL-1β, IL-6, and TNF-α [28]. Additionally, microglia-driven oxidative metabolism and ROS production have been implicated in axonal pathology development [25].

Oxidative stress plays a crucial role in structural protein damage, particularly affecting microtubules such as tubulin, leading to neurofilament hyperphosphorylation, a key driver of neurodegeneration [25]. Lipid peroxidation generates harmful by-products, including acrolein, MDA, and 4HNE, which modify carbonyl proteins, causing crosslinking, aggregation, and proteolysis resistance [29,30].

Several tauopathies have been linked to oxidative stress in TBI, particularly due to significant associations between beta-amyloid (βA) deposits and lipid peroxidation by-products [16]. A study by Castellani et al. [31] examined the potential mechanisms underlying tauopathy-like changes in TBI-affected brains, particularly in athletes and military personnel. However, limitations in their study prevented definitive conclusions.

Further research suggests that elevated ISOP concentrations in transgenic mouse models correlate with Alzheimer’s disease (AD)-like βA deposits, emphasising the role of lipid peroxidation by-products in βA accumulation and cognitive decline [32]. Additionally, repeated mild TBIs have been shown to trigger persistent oxidative stress responses lasting up to four months post-injury [32]. Thus, neuroinflammation and oxidative stress are likely to play a central role in sustaining secondary brain injury and should be considered in prognostic evaluations of TBI outcomes.

**Figure 1:**
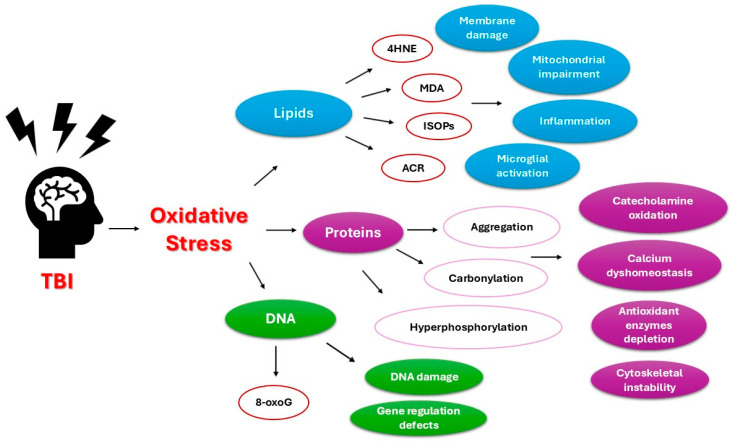
Fundamental pathways of oxidative stress effects in TBI (TBI—traumatic brain injury; DNA—deoxyribonucleic acid; 4HNE—4-hydroxynonenal; ISOPs—isoprostanes; MDA—malondialdehyde; ACR—acrolein; 8-oxoG—8-oxoguanine).

The interplay between ROS/reactive nitrogen species (RNS) and inflammatory cytokine activation has been well-documented in both neuroinflammation and TBI [33] (Figure 1). While inflammation is widely recognised for its role in brain oedema, its direct association with oxidative stress has only recently been established. Increasing evidence supports the notion that mitochondrial dysfunction, in combination with blood–brain barrier (BBB) breakdown, significantly contributes to secondary neuronal injury [34].

## 3. Antioxidant Defence in Traumatic Brain Injury

The body’s primary antioxidant defence system consists of enzymatic and non-enzymatic molecules that help neutralise ROS [14]. While the precise mechanisms by which antioxidant therapies mitigate TBI symptoms remain unclear, diets rich in antioxidants are often recommended post-injury to support recovery [35].

In TBI, ROS overproduction is counteracted by the antioxidant enzymatic cascade, which includes superoxide dismutase (SOD), catalase (CAT), and glutathione peroxidase (GPx). Similarly to other neurological pathologies, these enzymes play a crucial role in reducing oxidative stress. SOD, as the first-line defence against superoxide anions, has been extensively studied in conditions such as cancer, inflammatory diseases, ischemia, and neurodegenerative disorders. Studies in both human and animal models have reported decreased SOD levels in the extracellular environment following TBI, indicating impaired antioxidant response [34]. Due to its therapeutic potential, SOD has been explored as a target for innovative treatments [35]. For instance, research in concussed rats demonstrated that lecithinized SOD improved antioxidant activity and reduced brain oedema, highlighting its neuroprotective potential [36].

However, despite its crucial role, SOD’s catalytic activity produces hydrogen peroxide (H_2_O_2_), a less reactive but still potentially harmful ROS. H_2_O_2_ participates in various cellular processes, but excessive accumulation has been linked to diabetes mellitus, neurological disorders, vitiligo, and acatalasemia [37]. In the context of TBI, H_2_O_2_ disrupts potassium channel function and promotes apoptosis via Ca^2+^-dependent endonuclease activation in cerebral vascular smooth muscle cells [38,39]. The enzyme catalase (CAT) serves as the primary defence mechanism against hydrogen peroxide toxicity, converting it into harmless oxygen and water. In TBI treatment strategies, CAT’s potential has been studied through targeted endothelial vascular therapy, particularly via conjugated CAT administration with anti-ICAM-1 antibodies to enhance BBB protection [34,40].

Glutathione peroxidase also plays a key role in neutralising hydrogen peroxide, converting it into non-ROS molecules [41]. While some studies, including those by Fan et al. [42], have reported increased GPx levels following neuronal injury, research on its precise dynamics after TBI remains limited. Additionally, GPx activity has been shown to vary depending on the age at which the injury occurs, suggesting that age-related metabolic differences may influence antioxidant responses [41,42].

Beyond enzymatic antioxidants, low molecular weight antioxidants—such as vitamins C and E, N-acetyl-cysteine, flavonoids, carotenoids, resveratrol, coenzyme Q10, and omega-3 fatty acids—also contribute to reducing secondary injuries associated with TBI [43,44,45]. Studies by Di Pietro et al. [43] and Wu et al. [44] have demonstrated that vitamins C and E reduce mortality rates in neurotrauma patients by inhibiting lipid peroxidation and enhancing enzymatic antioxidant defence. Similarly, flavonoids and carotenoids have been found to lower oxidative stress in TBI by suppressing apoptosis and inflammation, reinforcing their potential role in neuroprotection and recovery [44,45].

## 4. Neuronal Damage Biomarkers and Oxidative Stress

### 4.1. Neuron-Specific Enolase

Neuron-specific enolase (NSE) is a brain-specific isoenzyme involved in glycolysis and is considered a key biomarker of neuronal damage [46]. In neuronal tissues, NSE is primarily expressed as homodimer structures (γ-γ), while glial cells predominantly express its heterodimeric form (α-γ) [47,48]. Notably, the γ-γ homodimers are resistant to chloride inactivation, which facilitates their intraneuronal localization [49]. In the cytosolic environment, NSE plays a crucial role in the glycolytic cycle [50,51]. However, in certain conditions, NSE can translocate to the cellular membrane, contributing to microglial activation and stimulating the production and release of pro-inflammatory cytokines [52].

NSE is widely recognised as a reliable molecular marker for TBI diagnosis and prognosis [53,54], as its translocation to cerebrospinal fluid (CSF) triggers inflammatory responses following neurodegeneration or neuronal injury [55,56,57]. Both homodimeric and heterodimeric forms, abundantly found in the grey matter, are also implicated in neuronal maturation [58]. However, NSE is not exclusively brain-specific; it is also expressed in neuroendocrine cells and can be detected in blood serum and CSF in malignancies [58]. Elevated NSE levels have been correlated with poor prognosis in several conditions [59], yet TBI management does not rely solely on NSE measurements [60].

Studies in rodent models indicate that serum NSE levels peak approximately six hours post-injury in cases of severe cortical impact [61]. In paediatric TBI, the timing of NSE peaks in CSF varies depending on the injury mechanism: 11 h post-injury in non-inflicted cases and up to 63 h in inflicted cases caused by blunt-force impacts on hard surfaces [62]. However, the magnitude of NSE peaks remains comparable between these injury types. Another study evaluating serum NSE as a predictive marker found that NSE levels typically peak within 12 h post-injury, with 48 h post-injury levels serving as an efficient outcome predictor [63]. A recent cohort study assessing the diagnostic performance of NSE in TBI reported significant fluctuations in NSE blood levels over the first 24 h, with the highest concentrations occurring between 9 and 16 h post-injury [64].

Over the past three decades, several studies have investigated the relationship between NSE levels and superoxide dismutase (SOD) antioxidant activity in various brain injuries. Some studies [26,60] analysing SOD and GPx kinetics in ischemic brain injury reported a delayed increase in SOD activity, peaking between two days and one week post-injury [26]. Additionally, GPx activity was found to be directly correlated with NSE levels. Gruener et al. [26] suggested that post-injury NSE serum levels and SOD activity may be linked, indicating that brain responses to damage and oxidative stress could be delayed due to defective signalling and vascular ischemia. Similar correlations were found in cerebral infarction patients [65]. However, further studies are required to fully elucidate the mechanisms underlying these biomarker interactions.

### 4.2. Ubiquitin C-Terminal Hydrolase L1

Ubiquitin C-terminal hydrolase L1 (UCH-L1) is a highly specialised enzyme expressed abundantly in the cells of the central nervous system and malignant processes, comprising up to 5% of total brain protein content [66]. In neurons, UCH-L1 is localised in both the cytosol and neuronal membranes, where it plays a key role in neuronal development and hypoxia-modulated apoptosis [67,68]. Additionally, UCH-L1 catalyses ubiquitination, targeting oxidised and misfolded proteins for degradation, which is crucial in synaptic function and DNA repair [67,69,70].

Following TBI, UCH-L1 expression is proportional to injury severity, with peak levels persisting for several days [70]. Unlike NSE, UCH-L1 blood levels peak within 8 h post-injury and decline significantly thereafter [64]. Papa et al. [71] demonstrated that UCH-L1 is detectable as early as one hour post-injury but loses diagnostic accuracy beyond 48 h due to rapid clearance. However, UCH-L1 is not a trauma-specific biomarker, as studies have shown its elevated levels in haemorrhagic shock and resuscitation models, making it less reliable for differentiating TBI from other injuries [72]. Deng et al. [73] also reported that serum UCH-L1 levels vary based on lesion type, with higher levels in mass lesions compared to diffuse injuries.

Notably, the CSF levels of UCH-L1 correlate with positive outcomes (survival), but its use as a biomarker for trauma severity is limited due to the invasive nature of CSF sample collection [69,74]. Mehta et al. [75] suggested that UCH-L1 alone is insufficient for predicting TBI recovery but may enhance diagnostic accuracy when combined with GFAP.

UCH-L1 also interacts with oxidative stress-related cellular byproducts, particularly lipid peroxides and ROS/RNS, which can modify cysteine residues at key structural sites [76]. Wang et al. [76] and Graham et al. [68] reported that oxidative stress-induced modifications of UCH-L1 contribute to neuronal degeneration in TBI, ischemic trauma, and neurodegenerative diseases. Additionally, UCH-L1 is a primary target of protein oxidation, with post-translational modifications linked to neuroinflammatory responses in AD and Parkinson’s disease [76].

### 4.3. S100 Calcium-Binding Protein B

S100B is a well-known damage-associated molecular pattern protein, also classified as an alarmin. Unlike calmodulin, a more ubiquitous calcium metabolism enzyme, S100B is cell-specific, modulated by environmental factors, and primarily localised intracellularly [77]. It is expressed in various vertebrate cell types, including astrocytes, oligodendrocytes, neural progenitor cells, Schwann cells, adipocytes, epithelial cells, and Leydig cells [78,79].

In the brain, where S100B is abundantly secreted, its main functions include neuronal protection, metabolic support, and modulation of neurogenesis and neuroplasticity [79]. However, under pathological conditions, S100B can be released extracellularly, making it a useful biomarker for tissue injury [80]. Following brain damage, glial cells rapidly secrete S100B into the bloodstream, where it is subsequently filtered and excreted by the kidneys [81]. The presence of S100B in blood or CSF is often indicative of BBB disruption and significant cerebral damage, as observed in TBI patients [81,82,83].

S100B has been extensively studied as a biomarker to reduce unnecessary neuroimaging after mild TBI [80]. While it is primarily found in the cytoplasm and nucleus, extracellular S100B concentrations remain extremely low under normal conditions, except in cases of BBB disruption and neuronal injury. A recent study suggested that adipocytes contribute to the rapid S100B peak in the bloodstream due to sympathetic activation post-injury, which promotes S100B release via cholinergic and adrenergic modulation [84]. Another mechanism of S100B secretion was described by Hermann et al. [85] in epileptic seizures, where Ca^2+^ and K^+^ levels modulate S100B secretion during excessive neuronal excitation.

Apart from brain injuries, S100B blood levels may also increase following bone fractures and muscle trauma [81,86]. During intense physical exertion, anaerobic glycolysis—which follows TBI-associated primary injury—correlates with mitochondrial dysfunction, oxidative stress, and neurodegeneration [87]. Animal studies have demonstrated that inhibiting S100B can attenuate TBI-induced lesions, oxidative stress, and microglial-mediated inflammation [88].

The diagnostic timeline of S100B remains controversial. A kinetic modelling study reported that S100B levels peak around 24 h post-TBI and remain detectable in blood and CSF for up to six days [79,89]. Rainey et al. [90] and Goyal et al. [79] found that serum S100B levels at 24 h post-TBI were predictive of poor outcomes (including mortality and unfavourable prognosis). However, Seidenfaden et al. [91] observed that serum S100B levels declined within one hour post-injury, prompting Haselmann et al. [92] to propose plasma S100B assessment as a faster, comparably effective diagnostic alternative.

Additionally, the extent of S100B release may correlate with post-TBI cognitive recovery [82,93,94]. A potential link between S100B and cognitive recovery has been suggested via the dopaminergic system [94,95]. SPECT imaging studies indicate that altered dopamine transporter levels in the striatum are associated with impaired processing speed and executive dysfunction in moderate-to-severe TBI patients [95]. Furthermore, astrocytic S100B regulation of dopaminergic neurons has been documented in Parkinson’s disease patients and animal models [96].

### 4.4. Glial Fibrillary Acidic Protein

Glial fibrillary acidic protein (GFAP) is a major cytoskeletal protein that forms intermediate filaments in mature astrocytes. Unlike vimentin, which is found in immature astrocytes, GFAP is abundant in mature astrocytes and serves as a key biomarker of astrocytic differentiation [97]. Studies indicate that GFAP is highly specific to brain injury, as it is absent in peripheral blood under normal physiological conditions [98,99]. It is released within one hour of brain injury and BBB rupture [100].

Compared to S100B, GFAP has a longer half-life, remaining detectable for up to 72 h post-trauma [98,101]. Like UCH-L1, GFAP is widely used as a biomarker for cranial trauma screening [98]. Its serum levels can change as early as 30 min post-injury, making it a potential early diagnostic marker. Papa et al. [71] identified GFAP as one of the earliest and most effective markers for detecting CT lesions. Conversely, a large study of diffuse TBI confirmed by CT imaging found that GFAP levels peaked at 20 h post-injury and declined over the following 72 h [102].

Korley et al. [103] assessed the potential of GFAP and UCH-L1 as TBI outcome predictors in a cohort of 2552 patients. Their study concluded that GFAP and UCH-L1 levels within the first 24 h post-injury were excellent predictors of mortality and unfavourable prognosis, though they were not reliable indicators of partial recovery within six months. Additionally, serum GFAP levels fluctuate based on TBI severity and mechanism, much like UCH-L1 [73].

Although S100B, GFAP, and UCH-L1 are all TBI biomarkers, GFAP has been particularly recognised for its diagnostic accuracy and ability to predict post-concussion imaging abnormalities [75,104]. Despite this, GFAP and UCH-L1 serve distinct functions due to their differing cellular origins and metabolic pathways [78]. UCH-L1, secreted by neurons, is involved in oxidised protein degradation, whereas GFAP, secreted by astrocytes, is released in response to astrocyte cytoskeletal injury. Thus, elevated GFAP levels may indicate focal mass lesions, whereas UCH-L1 is more indicative of diffuse injuries [75]. Mehta et al. [75] further reported that GFAP may be a specific biomarker for severe head injuries, as serum GFAP levels are not elevated in non-TBI conditions.

Regarding the interaction between GFAP and oxidative stress, studies have demonstrated that GFAP expression is upregulated by hydrogen peroxide accumulation. Morgan et al. [105] linked microglial activation, GFAP expression, and oxidative stress to age-related neurodegeneration. Similarly, Zhu et al. [106] found that hippocampal GFAP expression increases in response to excessive ROS accumulation, though the precise mechanisms remain unclear.

### 4.5. Neurofilament Proteins

Due to their high susceptibility to degeneration, neurons require extensive structural protection. Neurofilament proteins (NFPs) are neuron-specific intermediate filaments that, along with microtubules and microfilaments, form the neuronal cytoskeleton [107,108]. Five distinct types of NFPs have been identified, each of which can be affected by molecular alterations leading to pathological conditions. Under normal physiological conditions, small amounts of NFPs are present extracellularly, in the CSF and blood. However, their elevated concentrations in bodily fluids serve as reliable biomarkers of neuronal injury [109,110].

In rodent models of neuronal injury, NFP levels peak a few days post-cranial impact, correlating with sensorimotor deficits [111]. Light chain NFP levels typically peak within seven days post-injury and can remain elevated for months or even years. Clinically, light chain NFP is a valuable diagnostic biomarker, with its levels being detectable from emergency admission up to 12 days post-injury [112]. Another study demonstrated that plasma light chain NFP levels peak between 10 days and 6 weeks post-injury, persisting for up to a year [113]. Andersson et al. [114] observed that CSF light chain NFP levels progressively increase over the first two weeks post-TBI, with some patients showing elevated levels up to eight months post-injury, but not beyond five years, as reported by Newcombe et al. [115] and Shahim et al. [116].

Shahim et al. [112] further highlighted that light chain NFP levels in serum and CSF serve as strong prognostic markers for TBI severity and outcome. Among all TBI biomarkers, light chain NFP has shown the greatest prognostic value, particularly when measured from CSF samples [111]. Yuan and Nixon [109] established a correlation between light chain NFP levels, axonal diameter, and both sensorimotor and cognitive impairment. However, due to the invasive nature of CSF collection, blood-based assays such as Western blotting and immunohistochemistry staining are preferred for TBI and spinal cord injury diagnostics [112,117].

NFP structural changes can result from multiple molecular pathways, including oxidative stress [117]. Recent studies indicate that ROS trigger oxidation of peptides and lipids, leading to loss of function and ferroptosis [4,118,119]. Gelinas et al. [30] demonstrated that oxidative stress modifies the secondary structure of NFPs, transitioning from α-helices to β-sheets and random coils. They hypothesised that ROS-induced NFP structural changes promote cytoplasmic inclusion formation, a hallmark of neurodegeneration [30]. Similar findings were reported by Gowthami et al. [120] and Bielanin et al. [121]. Further research is needed to fully elucidate the functional consequences of oxidative modifications in structural neuronal proteins.

### 4.6. Myelin Basic Protein

Myelin is a complex multilamellar structure composed of cholesterol, phospholipids, glycolipids, and several proteins, including proteolipid protein, myelin basic protein (MBP), and myelin protein zero [122,123]. Unlike other neuronal components, myelin sheaths are not synthesised by neurons, but rather by oligodendrocytes in the CNS and Schwann cells in the peripheral nervous system [123].

MBP, the second most abundant myelin protein, plays a critical role in myelin sheath adhesion, linking it to the cytosol, cytoskeleton, and plasma membrane [124]. Additionally, MBP is essential for extracellular signalling, axonal insulation, and efficient electrical impulse transmission [125]. Despite its diagnostic significance in TBI and neurodegeneration, MBP is challenging to detect due to its structural variability and sensitivity [126]. In both TBI and multiple sclerosis, increased MBP levels in CSF indicate severe brain injury [125]. However, its low disease specificity has resulted in limited data on MBP variations in serum and CSF of TBI patients. Moreover, the precise pathological mechanisms involving MBP and calcium metabolism remain poorly understood [125]. In contrast, Bohnert et al. [127] demonstrated that the assessment of MBP from CSF effectively distinguishes lethal TBI from other causes of death.

The timing of MBP level fluctuations post-TBI has been explored in several studies. Su et al. [128] reported that CSF levels of MBP in paediatric patients remained significantly elevated for up to five days post-injury compared to controls. Additionally, MBP concentrations differed significantly in children who had sustained a TBI over a year before assessment versus those with recent injuries (<1 year prior). Multiple studies have also shown that CSF levels of MBP vary depending on the type of head trauma (e.g., abusive vs. non-abusive injuries) [128,129,130]. Similarly, Singh et al. [131] conducted a prospective study demonstrating that serum levels of MBP serve as a useful prognostic marker in mild-to-moderate head injury. Oehmichen et al. [132] identified MBP-positive macrophages in the brains of adult TBI patients as early as 17 h post-injury.

Regarding the interaction of MBP with oxidative homeostasis, Businaro et al. [133] described a mechanism through which MBP regulates calcium-dependent activation of heme oxygenase-1 in astroglia. However, beyond this, the impact of oxidative stress on MBP structure and function remains largely unexplored, presenting an avenue for future research.

## 5. Neurodegeneration Biomarkers and Oxidative Stress

### 5.1. Tau Protein

Tau is a microtubule-associated protein that plays a crucial role in neuronal stability [133,134]. It is involved in tubulin assembly, axonal integrity of unmyelinated neurons, and cortical interneurons [133,134,135,136]. Additionally, tau contributes to signal mediation, synaptic activity, cell proliferation, neurodevelopment, and neuroplasticity [31,135].

Tau activation occurs primarily via phosphorylation; however, the most common changes in tau involve post-translational modifications. Acetylation and hyperphosphorylation of tau promote the formation of neurofibrillary tangles (NFTs), a hallmark of neurodegeneration seen in AD and other tauopathies [135]. Recent research has identified a similar mechanism of NFT formation in TBI, suggesting overlapping pathophysiological processes between TBI and AD [137,138]. Furthermore, studies have demonstrated that tauopathies exhibit prion-like behaviour, where tau aggregates spread across the brain, and that anti-tau antibodies can reverse motor deficits in experimental models [139,140].

Tau protein cleavage by caspases has been linked to cognitive decline, as seen in both AD and TBI [140,141,142]. Rizzi and Grinberg [143] recently described the role of caspase enzymes in cleaving tau at its C- and N-terminal ends, resulting in mitochondrial dysfunction and impaired axonal transport. These tau fragments contribute to neuronal injury, NFT formation, and amyloid plaque development.

Chronic traumatic encephalopathy (CTE), a long-term consequence of repeated TBI, shares molecular and clinical characteristics with dementia [141]. Rubenstein et al. [141] reported that tau protein levels in serum increase within 1 to 6 h post-injury in TBI rodent models, making it a potential early biomarker. Clinical studies have confirmed that serum tau levels rise immediately post-TBI, peak around 48 h, and decline over the following week, with concentrations correlating with injury severity [144,145]. However, Shahim et al. [146] noted significant variability in tau levels across different time points (30, 60, and 90 days, as well as 1 to 5 years post-TBI), emphasising the challenge of using tau as a long-term prognostic marker.

Animal studies indicate that tau protein expression varies depending on injury mechanism. Single vs. repetitive blast injury models exhibit distinct tau phenotypes, suggesting that injury redundancy influences tau pathology [147]. Additionally, the oxidative stress hypothesis of ageing provides insight into tau pathology in TBI. ROS accumulation, a shared mechanism in ageing and neurodegeneration, contributes to tau aggregation and NFT formation, reinforcing the link between oxidative stress and tauopathies [138].

### 5.2. Beta Amyloids

Beta amyloids (βA) are peptides derived from amyloid precursor proteins (APP) via proteolysis by secretases [148]. Their metabolism occurs primarily in neuronal endosomes and lysosomes, where proteases degrade APP into βA peptides [149].

Although multiple pathways contribute to βA degradation, some βA escapes clearance and is drained into CSF, lymphatic fluid, or blood. This degradation system helps prevent βA accumulation, which otherwise promotes ageing and neurodegeneration. The formation of senile plaques, composed of misfolded hyperphosphorylated tau and βA peptides, is a defining feature of dementia and AD, contributing to cognitive impairment and neuronal loss [150].

Studies suggest that βA accumulation and NFT formation follow similar pathways in AD and repetitive TBI [135,151,152]. In addition, axonal injuries correlate with βA build-up, as βA-degrading enzymes accumulate in damaged axons [135,152]. One key difference between TBI-associated neurodegeneration and AD is the timing of βA and NFT formation. In TBI, NFTs and βA deposits in CSF or serum can be detected within hours post-injury [152]. Moreover, studies have shown that TBI induces βA1-42 accumulation, the most toxic βA variant, highlighting parallels with familial AD and diffuse axonal injury [153,154,155,156,157]. The disruption of APP axonal transport remains the primary cause of βA accumulation in injured neurons [158].

In the search for biomarkers of neurodegeneration, Saha and Sen [135] demonstrated that CSF levels of tau and βA serve as predictors of NFT formation. However, conflicting study results highlight the challenges of using molecular markers for TBI-associated neurodegeneration due to variability in measurement timing [152]. Recent findings indicate that βA aggregation begins soon after TBI, with deposits observed in brain tissue and leptomeningeal arteries within hours to 13 days post-injury [159]. Romero-Tirado et al. [160] linked βA levels to mortality risk in TBI patients, concluding that βA is one of the few biomarkers predictive of injury-related death. Marklund et al. [161] further reported that CSF levels of βA differ significantly between focal and diffuse axonal injuries, suggesting that injury type influences βA accumulation patterns.

The oxidative stress hypothesis of AD suggests that βA plays a role in oxidative damage and inflammation [126]. Beta amyloid deposits promote ROS accumulation and metal ion chelation, leading to hydrogen peroxide formation [32,156,157]. Excess ROS has been shown to trigger βA accumulation within lysosomal vesicles, exacerbating neuronal damage [28,157].

Pro-inflammatory cytokines, including IL-1, IL-6, and TNF-α, are implicated in βA accumulation, neuronal loss, and cognitive decline [157]. The association between βA and oxidative stress in TBI has been extensively investigated. Johnson et al. [152] reviewed TBI animal models where βA plaques were identified, suggesting that oxidative stress upregulates γ-secretase activity, leading to excessive βA production and aggregation of toxic amyloid peptides. Given the significant role of oxidative stress in TBI pathogenesis, βA-mediated oxidative damage remains a crucial target for future therapeutic strategies [34].

## 6. Genetic Biomarkers and Oxidative Stress

### 6.1. Transactive Response DNA-Binding Protein-43

Transactive response DNA-binding protein 43 (TDP-43), encoded by the TARDBP gene, is a nuclear ribonucleoprotein that plays a role in gene regulation and RNA metabolism across various tissues [158,159]. Mutations in TARDBP have been associated with amyotrophic lateral sclerosis (ALS), frontotemporal dementia (FTD) with parkinsonism, and AD [160,161,162]. The mature TDP-43 protein regulates transcription [163], messenger RNA (mRNA) stability, and miRNA processing [164].

Post-translational modifications such as ubiquitination, phosphorylation, and abnormal cleavage contribute to TDP-43 aggregation, a key feature of neurodegenerative diseases [165]. Additionally, Jo et al. [166] demonstrated that zinc ions facilitate TDP-43 accumulation and promote amyloid-like aggregates in vitro.

The distinction between TDP-43-related neurodegenerative disorders and TDP-43 neuropathological changes in TBI was noted by Chen et al. [167]. In single TBI cases, TDP-43 is overexpressed but not significantly altered by post-translational modifications such as hyperphosphorylation. However, recent studies suggest that TDP-43 dysregulation contributes to the progression of TBI-related neurodegeneration, particularly in cases with AD-like traits [168]. Some authors have proposed that TDP-43 loss of function may drive hyperphosphorylated tau accumulation, further linking TBI and AD pathogenesis [169].

Interestingly, TDP-43 structural and functional abnormalities have been reported in patients with cognitive impairment, particularly those exhibiting executive dysfunction, a hallmark of FTD rather than AD [170]. This pattern of frontal and motor deficits closely resembles the cognitive impairments observed in TBI, further strengthening the TDP-43-TBI connection. In line with this, a murine model of cortical injury exhibited altered TDP-43 function in both brain and spinal cord tissues [171]. Similarly, Gao et al. [172] linked TDP-43 overexpression to neurodegeneration in a TBI mouse model, demonstrating that both wild-type and knockdown phenotypes exhibited neurocognitive impairments. Neuroinflammatory responses in TBI animal models were also shown to induce TDP-43 overexpression, leading to AD-like neurodegeneration [172].

Furthermore, Janković et al. [173] suggested that the mechanism of TBI (e.g., single vs. repetitive trauma) dictates TDP-43 alterations, possibly through a microglial-mediated neuroinflammatory response. Recent studies have also identified TDP-43-specific encephalopathy, known as limbic-predominant age-related TDP-43 encephalopathy (LATE), which mimics AD-like cognitive decline and is characterised by phosphorylated TDP-43 aggregates in the neuronal cytoplasm [174,175,176].

Despite these findings, the direct link between TDP-43 accumulation and TBI-induced neurodegeneration (e.g., chronic traumatic encephalopathy, CTE) remains under investigation. Nevertheless, increased TDP-43 levels have been associated with ALS, repetitive concussions, and other forms of head trauma [169].

Anderson et al. [177] demonstrated that nucleoporin dysfunction contributes to TDP-43 mislocalization, aggregation, and accumulation in the brains of both TBI-exposed Drosophila models and CTE patients, highlighting its role in long-term TBI consequences.

The time course of TDP-43 alterations post-injury has been studied in various models. Bjorklund et al. [175] reported that TDP-43 aggregates accumulate in the frontotemporal cortex of mice from 7 days to 6 months post-TBI. Janković et al. [173] found that TDP-43 expression varies depending on injury mechanism (e.g., single vs. repetitive trauma) and is closely tied to microglial activation. Additionally, Martin et al. [178] observed that TDP-43 aggregates may form distantly from the primary injury site, persisting for up to 6 months in human iPSC-derived motor neurons.

In military service members with blast-related TBI, Heyburn et al. [171] noted that TDP-43 levels were significantly elevated in repetitive TBI cases, particularly in those who later developed frontotemporal dementia (FTD).

The role of TDP-43 in oxidative stress has been explored through the formation of stress granules following oxidative injury. Colombrita et al. [179] demonstrated that oxidative stress enhances TDP-43 recruitment into ribonucleoprotein complexes, disrupting protein synthesis and impairing mRNA translation, which contributes to motor neuron degeneration. Additionally, cytoplasmic TDP-43 aggregates have been shown to interfere with mRNA transport and axonal function, further exacerbating oxidative stress-induced neuronal dysfunction [180].

### 6.2. Micro RNA

MicroRNAs (miRNAs) are highly conserved, non-coding RNA molecules that regulate gene expression at the post-transcriptional level. Micro RNAs can induce messenger RNA degradation or modulate transcription and translation [181,182]. Stored in small intracellular vesicles, miRNAs are increasingly recognised as biomarkers of disease, as they are detected in exosomes and bodily fluids under pathological conditions [182].

In neurons, miRNAs regulate differentiation, proliferation, apoptosis, and metabolism, making them important biomarkers of neural loss, synaptogenesis, and neuroplasticity [181,182,183,184]. Given their ability to cross the BBB and participate in both primary and secondary injury responses, miRNAs have emerged as key biomarkers in TBI [185,186].

Recent studies have detected multiple miRNA species in the blood serum of TBI patients, with distinct expression patterns at different post-injury time points [183,184,185,186]. The kinetics of miRNA expression span CSF, blood, and saliva, with changes occurring in both primary and secondary injury phases [185,186,187,188,189,190]. Significant alterations in key miRNA expression have been reported in both clinical and animal model studies, starting as early as 1 h post-TBI and persisting for up to 7 days [191,192,193,194,195,196,197].

Di Pietro et al. [191] found that miRNA expression changes first become detectable in saliva, though they diminish by 120 h post-injury. Musso et al. [198] reported that serum miRNA levels tend to normalise within six months post-TBI. Moreover, the study brings additional evidence regarding the changes in expression in some miRNA species. In this way, Musso et al. [198] reported that miRNAs 150-5p, 132-3p, and 23b-3p levels were significantly lower in TBI patients’ sera, as compared to healthy controls at baseline. As miRNA 150-5p is known to contribute to neuroinflammatory response to brain injury, being an important marker of major trauma onset, while miRNA 132-3p and 23b-3p are implicated in neuronal repair and apoptosis, it was suggested that they are potent biomarkers for TBI outcomes [198,199,200]. On the other hand, several studies have also reported significant association between these miRNA species and oxidative stress pathways, as, for example, miRNA 23b-3p was shown to contribute to oxidative stress-modulated apoptosis inhibition and protect against Alzheimer’s disease [201,202]. Similar functions were reported for both miRNA 150-5p and 132-3p, including regulation of endoplasmic reticulum stress, oxidative stress-modulated apoptosis and inflammatory response [200,203].

Extensive studies have shown that miRNAs participate in the regulation of oxidative stress and neuroinflammation in TBI [204]. miRNAs are also implicated in cerebral blood flow regulation [205]. As noted by Konovalova et al. [206], miRNA profiles are disease-specific, distinguishing TBI from other neurological disorders.

Key miRNAs differentially expressed in TBI patients include miR-9, miR-16-5p, miR-21-5p, miR-130a, miR-155, miR-451, and miR-23a-3p, which correlate with injury severity and mortality [186,188]. Notably, miR-9 and miR-451 were implicated in hippocampal inflammatory responses following controlled cortical impact in mice [207].

Zhu et al. [208] extensively reviewed the overlap between miRNAs in mild TBI and post-traumatic stress disorder, particularly highlighting miR-144’s role in oxidative stress regulation.

Some miRNAs directly modulate ROS production. Saha et al. [209] identified a subset of “redoxi-miRNAs” involved in oxidative stress control. Xu et al. [210] demonstrated that miR-27b is involved in iron-induced oxidative stress, neuroinflammation, and apoptosis, targeting NRF2 mRNA and suppressing SOD1 gene expression, exerting pro-oxidant effects. Conversely, miR-124 has an antioxidant function, inhibiting pro-oxidant pathways and promoting post-TBI recovery [211].

### 6.3. Long Non-Coding RNA

Long non-coding RNAs (lncRNAs) are a diverse class of RNA molecules that do not encode proteins [212,213]. Instead, lncRNAs play crucial roles in cell survival, proliferation, and genetic stability by modulating gene expression and chromatin architecture [212]. Some lncRNAs also function as regulators and competitors of microRNA (miRNA) expression, influencing post-transcriptional gene regulation [214]. Similarly to mRNA, lncRNAs are transcribed by RNA polymerase II [214,215].

Recent studies have identified high levels of lncRNA expression in the mammalian brain, where they contribute to neocortical development and neuronal function [212,215]. It is estimated that more than 40% of all lncRNA molecules in the body are produced by brain cells [214,216]. Among their many functions, the most significant neurological role of lncRNAs is the regulation of gene expression, particularly in pathways related to synaptogenesis, neurodevelopment, and neurodegenerative disorders [217].

Beyond their physiological roles, lncRNAs have been implicated in neuropathological conditions. Some lncRNAs are transcribed from genomic regions near genes involved in synaptogenesis and AD pathogenesis, suggesting a potential role in neurodegenerative processes [214]. While their precise contribution to disease mechanisms remains under investigation, lncRNAs may regulate or even exacerbate neurodegenerative pathologies, including those associated with TBI.

In the context of TBI, lncRNAs are emerging as key mediators of the neurological response to brain trauma. Rodent models have shown that neocortical lncRNA expression is significantly altered within 24 h post-TBI and may correlate with astrocyte apoptosis via TNF-α upregulation [218,219]. Other animal model studies have detected lncRNA expression changes within hours post-injury, with alterations persisting for up to 14 days [218,219]. Patient studies have similarly demonstrated that lncRNA levels increase significantly post-TBI, are detectable within 24 h, and may persist for several days [220,221,222,223,224].

Further analysis of TBI patients suggests that lncRNAs play a role in post-TBI neuroinflammation by stimulating microglial activity, particularly in concussed brain regions, while sparing surrounding tissues [213]. Several studies also suggest that certain lncRNA species contribute to post-TBI recovery, helping to delay secondary injury, suppress tumorigenesis, and promote tissue regeneration [219].

An interesting example of TBI-associated variation in lncRNA expression was recently reported by Patel et al. [225]. Despite very little being known about the implication of lncRNA VLDLR-AS1 in brain damage, the study found significant correlations between lower expression of lncRNA VLDLR-AS1 in the sera of the patients that experienced repeated TBIs, as compared to healthy controls. The study discussed the implication of this lncRNA species to modulating cellular stress response in some malignant processes but suggested that its contribution to brain processes is still unclear. However, the evidence in favour of lncRNA VLDLR-AS1 as a valuable biomarker of TBI diagnosis is significant (with 90.89% power at the 5% significance level, by nQuery-based power analysis).

Although research on lncRNA involvement in oxidative stress regulation post-TBI remains limited, studies in other tissue damage models, such as hepatic injury, renal failure, and lung cancer, have described lncRNA interactions with oxidative pathways, as reviewed by Zhang et al. [226]. Some evidence suggests that lncRNAs also contribute to oxidative stress regulation in some pathologies of the central nervous system [227].

Animal models of hydrogen peroxide-induced optic nerve injury suggest that lncRNAs modulate oxidative stress responses in TBI, similar to their role in other CNS disorders [228,229,230]. Given that oxidative stress is a major driver of secondary injury post-TBI, lncRNAs may exert neuroprotective effects by activating key signalling pathways such as JAK/STAT3 and JNK, which regulate cell survival and inflammation [227,228,229,230].

Another potential mechanism linking TBI, oxidative stress, and lncRNAs involves intracranial haemorrhage, brain swelling, and neuroinflammation. Certain lncRNAs—already implicated in autoimmune diseases and malignancies—may interact negatively with miRNAs involved in oxidative stress regulation and BBB permeability, potentially worsening post-TBI complications [231,232].

As new lncRNA species continue to be identified, additional molecular players in TBI pathology and recovery are being uncovered. For instance, the antisense RNA of brain-derived neurotrophic factor functions as a BDNF gene expression suppressor, preventing the repair and neuroprotective effects of BDNF in both in vivo and in vitro models of hypoxia-induced brain injury [233].

Given the well-established link between oxidative stress and neuroinflammation in TBI [12,30], it is likely that lncRNAs influence oxidative responses beyond simple ROS regulation, engaging in complex molecular mechanisms that modulate both neurodegeneration and neurorepair. Future research should further investigate these interactions, potentially identifying novel therapeutic targets for post-TBI intervention.

## 7. Challenges and Future Perspectives

Despite these advances, several challenges remain regarding fully integrating biomarker-based diagnostics and oxidative stress modulation into clinical practice. Increased heterogeneity in TBI presentation, including individual differences in TBI manifestations, variations in injury severity, mechanism, and affected brain regions, could complicate the standardisation of biomarker interpretation. Moreover, as many TBI biomarkers fluctuate as time passes from the injury, the optimal window for biomarker assessment leading to efficient diagnosis and prognosis could often be missed. Another important challenge that needs further addressing is that oxidative stress is a non-specific factor that contributes to the development and escalation of TBI-related brain damage and secondary injuries. Studies have previously demonstrated that oxidative stress is implicated in similar co-occurrent effects in other neurodegenerative and inflammatory disorders, limiting its specificity as a standalone diagnosis indicator. In this context, while preclinical studies provide compelling insights into TBI-related oxidative mechanisms, clinical validation and large-scale biomarker studies are still needed to bridge the gap between experimental models and real-world applications.

The integration of oxidative stress biomarkers with neuroimaging, electrophysiology, and advanced biochemical assays holds promise for developing personalised diagnostic and therapeutic strategies for TBI patients. Combining multi-omics approaches (including proteomics, genomics, and metabolomics) with machine learning algorithms could enhance predictive models for TBI prognosis, enabling tailored interventions based on individual biomarker profiles.

## 8. Conclusions

Traumatic brain injury (TBI) is a highly complex neuropathological condition that results from various forms of cranial impact. The functional impairments caused by TBI are often transiently masked by acute clinical symptoms, making it challenging to assess long-term neurological and cognitive consequences. As a result, current diagnostic and prognostic approaches may fail to capture the full extent of TBI-induced brain dysfunction, particularly in mild or repetitive injuries.

The ongoing validation of molecular biomarkers has led to a deeper understanding of TBI pathophysiology, revealing intricate biochemical and functional interactions within the injured brain. Among these, oxidative stress has emerged as a critical yet non-specific factor that plays a dual role in both injury progression and recovery mechanisms. While oxidative stress contributes to neuronal damage, inflammation, and apoptosis, it also activates repair pathways, highlighting the dynamic and context-dependent nature of its effects in TBI.

Recent studies have provided strong evidence that oxidative stress and molecular biomarkers are closely interconnected, influencing disease progression, recovery potential, and patient outcomes. Key biomarkers such as NSE, S100B, GFAP, UCH-L1, and tau protein have demonstrated significant correlations with oxidative stress-mediated neuronal damage. Additionally, novel molecular players, including lncRNAs and miRNAs, have been identified as potential regulators of oxidative pathways, further expanding our understanding of secondary injury mechanisms.
